# Gut Microbiota Metabolites Differentially Release Gliotransmitters from the Cultured Human Astrocytes: A Preliminary Report

**DOI:** 10.3390/ijms24076617

**Published:** 2023-04-01

**Authors:** Michał Seweryn Karbownik, Paulina Sokołowska, Edward Kowalczyk

**Affiliations:** Department of Pharmacology and Toxicology, Medical University of Lodz, Żeligowskiego 7/9, 90-752 Lodz, Poland

**Keywords:** gut microbiota, probiotics, metabolites, butyrate, indole-3-propionic acid, gliotransmitters, ATP, astrocytes, antidepressant, mechanism of action

## Abstract

Butyrate and indole-3-propionic acid represent the CNS-available gut microbiota metabolites exhibiting potentially beneficial effects on human brain function and being tested as antidepressants. Astrocytes represent one of the putative targets for the gut metabolites; however, the mechanism of action of butyrate and indole-3-propionic acid is not well understood. In order to test this mechanism, a human astrocyte cell-line culture was treated with the compounds or without them, and the supernatants were collected for the analysis of ATP and glutamate gliotransmitter release with the use of luminescent and fluorescent methods, respectively. A 10-min incubation of astrocytes with 1–5 mM butyrate increased the ATP gliotransmitter release by 78% (95%CI: 45–119%), *p* < 0.001. The effect was found to be mediated by the cytosolic Ca^2+^ mobilization. Both 10-min and 24-h treatments with indole-3-propionic acid produced no significant effects on the release of gliotransmitters. The results for glutamate release were inconclusive due to a specific glutamate release pattern discovered in the tested model. This preliminary report of butyrate-induced ATP gliotransmitter release appears to provide a novel mechanistic explanation for the beneficial effect of this gut microbiota metabolite on brain function; however, the results require further evaluation in more composed models.

## 1. Introduction

Human intestines are inhabited by trillions of microorganisms being collectively called the gut microbiota, that constitute an indispensable component of the human holobiont system [1,2]. The quality of gut microbiota and their interaction with the host appear to significantly impact human development and adult physiology, also in terms of neuropsychological functioning and relevant pathology [3]. The mechanisms underlying these effects are outlined by the gut–brain axis (GBA) signaling. The GBA involves multiple pathways and messengers such as the vagus nerve [4], the hypothalamic–pituitary–adrenal axis [5], microbiota-derived neurotransmitters and their precursors [6,7], neurotrophic factors [8], the immune system [9], and bacterial metabolites [10]. The latter include a set of thousands of specific chemical compounds that reach distant tissues, including the central nervous system (CNS), and play a role in the homeostasis of the human organism [1,10,11].

Among the metabolites produced by the human gut microbiota, butyrate (BUT) and indole-3-propionic acid (IPA) are of special interest to neuropsychiatry as both are absorbed from the gastrointestinal tract, penetrate the blood–brain barrier [11], and are capable of interacting with the brain components. BUT is one type of the body’s short-chain fatty acids (SCFA), which constitute the family of major gut microbial metabolites [12]. SCFA are produced in the colon largely by the bacterial fermentation of dietary fibers and resistant starch [12,13]. BUT may interact with the brain cells by binding to free fatty acid receptors (FFAR) [10], among others, thus contributing to anti-neuroinflammatory, and consequently antidepressant and pro-cognitive, effects [14,15,16,17,18,19,20,21,22,23]. IPA is a tryptophan derivative, which is present in the human body only thanks to microbe-mediated metabolism [24]. IPA binds to aryl hydrocarbon receptor (AhR) which regulates the transcription of genes [11], leading to neuroprotective and cytokine-suppressing effects in the astrocytes [22,25]. However, the molecular mechanisms of beneficial actions of both BUT and IPA are not fully understood.

Astrocytes represent an abundant cell type in the CNS, playing a critical role in brain function [26]. Astrocytes help maintain the homeostasis of neuronal synapses. They respond to neurotransmission by releasing their own substances, called gliotransmitters, to control neuronal excitability and synaptic information-processing, thus contributing to synaptic plasticity [27], and the impairment of gliotransmission may contribute to brain pathology [28,29]. Among the well-studied gliotransmitters are adenosine 5′-triphosphate (ATP) and glutamate (Glu). Astrocyte-released ATP controls purinergic neurotransmission in a long-lasting manner. It modulates enduring changes in glutamatergic and γ-aminobutyric acid synaptic efficacy [30]. Impaired ATP release from brain astrocytes may be a cause of depressive-like behavior in several animal models [31,32,33], and ATP administration or the induction of its specific release from astrocytes results in rapid antidepressant-like effects [34]. Glu is best known as a neurotransmitter, and the astrocytes uptake synaptically released Glu to optimize neuronal function and prevent excitotoxicity [35]. However, recent scientific interests have also focused on Glu as a gliotransmitter [27,35]. Although the clinical significance of the astroglial-released Glu remains to be elucidated, it appear to synchronize excitatory and inhibitory neuronal firing, thus contributing to brain homeostasis [35].

BUT and IPA have been found to influence vitally important astrocyte functions [22,36,37,38]. However, it is unknown whether the metabolites are capable of modulating the gliotransmitter release as well. Such a hypothesis is mechanistically justified, as the activation of the molecular targets for BUT and IPA—which are expressed in astrocytes [39,40]—has the potential to increase cytosolic Ca^2+^ levels [41,42], the major mediator of the release of ATP and Glu from the cells [27]. Based on this hypothesis, the aim of the present study is to evaluate the effects of BUT and IPA on ATP and Glu gliotransmitter release from cultured human astrocytes and to check whether the mechanism is dependent on cytosolic Ca^2+^ mobilization [43]. Here, we present the preliminary results of the research.

## 2. Results

### 2.1. The Effect of the Tested Gut Metabolites on Cell Viability

Before the main analyses were performed, the astrocyte cells’ viability was determined under the influence of the tested gut microbiota metabolites to exclude their toxicity. There were no significant effects of BUT (up to 25 mM) and IPA (up to 0.25 mM) on astrocytes viability after 24-h treatment, and the mean viabilities of the cells treated with BUT and IPA ranged from 92% to 105% of the control cells.

### 2.2. ATP and Glu Release Dynamics

**Figure 1:**
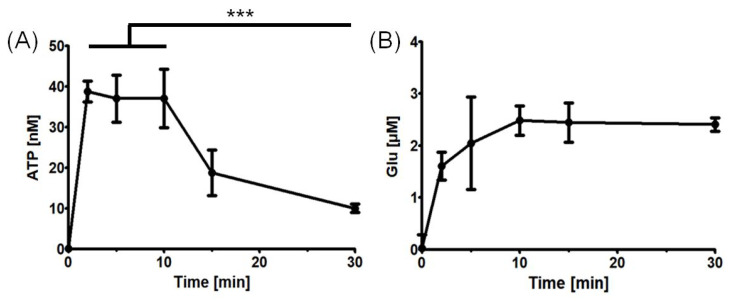
ATP and Glu release dynamics from the astrocyte cultures. (**A**) ATP release after cell wash and fresh experimental medium addition at various time points. ATP concentration was found to be significantly reduced at 30 min as compared with at 2–10 min (Welch’s *t*-test, *** *p* < 0.001). (**B**) Glu release after cell wash and HEPES-buffered solution addition at various time points. The points represent the mean estimates, whereas the whiskers indicate the standard errors of means. Number of biological replicates n ≥ 4 for each time point.

Then, in order to validate the research model, the dynamics of ATP and Glu release were investigated without the tested gut metabolites addition. The release of ATP and Glu from the astrocyte culture was found to be time-dependent. ATP was released rapidly [44], with maximum extracellular concentrations reached at 2–10 min, and with the significant decrease afterwards likely due to the action of ectonucleotidases [45]. On the other hand, extracellular Glu accumulated during the first 10 min and appeared not to decrease later on (Figure 1).

### 2.3. ATP and Glu Release in Response to Control Compounds

**Figure 2:**
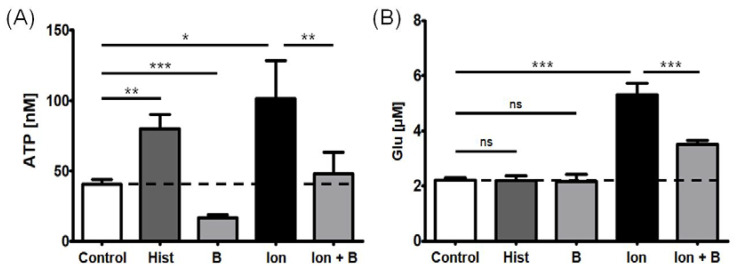
Effect of 10-min treatment with positive controls (histamine 100 μM and ionomycin 5 μM) and a negative control (BAPTA-AM 50 μM 30-min pre-treatment) on (**A**) ATP and (**B**) Glu release from the astrocyte cultures. The effect of BAPTA-AM was tested in the presence and absence of ionomycin. The bars represent the mean estimates, whereas the whiskers indicate standard errors of means. Dashed lines indicate the level of ATP and Glu released in control conditions (fresh experimental medium or HEPES buffered Ca^2+^-containing solution, respectively). The number of biological replicates n ≥ 9 for each condition. One-way analysis of variance with post hoc Dunnett’s test, significance level α = 0.05. * *p* < 0.05, ** *p* < 0.01, *** *p* < 0.001. ns—not significant, Hist—histamine, B—BAPTA-AM, Ion—ionomycin, Ion + B—ionomycin following 30-min pre-incubation with BAPTA-AM.

In order to further determine the nature of ATP and Glu gliotransmitter release from the tested astrocytes, several control compounds were used. Ionomycin—a Ca^2+^ ionophore that increases the cytosolic Ca^2+^ concentration mainly by facilitating the transport of the ions across the plasma membrane and from intracellular stores [46]—significantly increased ATP and Glu release; both effects were attenuated by the concomitant presence of an intracellular Ca^2+^ chelator [47]—BAPTA-AM. However, histamine—a molecule that mobilizes cytosolic Ca^2+^ in response to histamine H_1_ receptor activation, in a mode mediated by phospholipase C (PLC) and inositol 1,4,5-trisphosphate (IP_3_) [48]—evoked an enhanced ATP release, but not Glu, in the tested astrocyte cell line. Furthermore, BAPTA-AM could significantly reduce baseline ATP release, but not Glu release, in the cells treated with no Ca^2+^-mobilizing agents (Figure 2).

### 2.4. The Effects of the Tested Gut Metabolites on ATP and Glu Release

**Figure 3:**
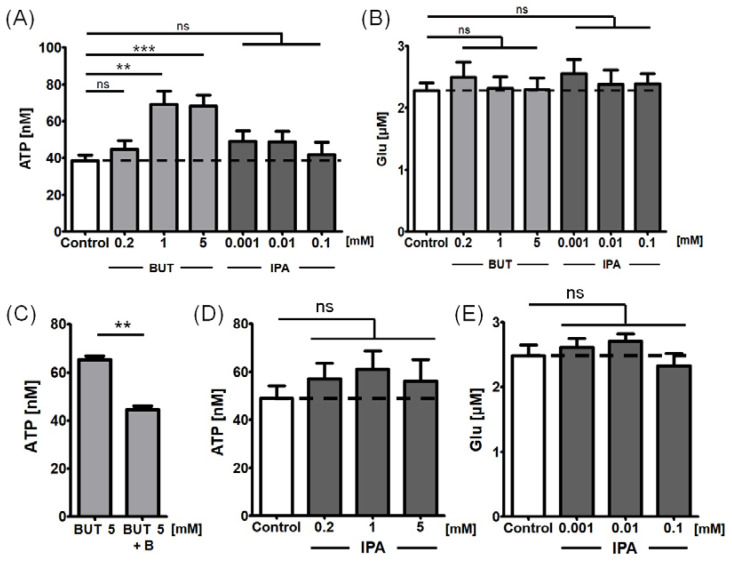
Effects of BUT and IPA on ATP and Glu release from the cultured astrocytes. (**A**) Effect on ATP release following 10-min treatment. (**B**) Effect on Glu release following 10-min treatment. (**C**) Attenuation of the BUT effect on ATP release by 30-min pre-incubation with BAPTA-AM 50 μM. (**D**) Effect on ATP release following 24-h pre-treatment with IPA. (**E**) Effect on Glu release following 24-h pre-treatment with IPA. The bars represent the mean estimates, whereas the whiskers indicate standard errors of means. Dashed lines indicate the levels of ATP and Glu release in control conditions (fresh experimental medium or HEPES buffered Ca^2+^-containing solution, respectively). Number of biological replicates n ≥ 9 for each condition. One-way analysis of variance with post hoc Dunnett’s test, significance level α = 0.05. ** *p* < 0.01, *** *p* < 0.001. ns—not significant, B—BAPTA-AM.

In concentrations of 1–5 mM, 10-min incubation with BUT significantly increased the release of ATP from the astrocyte cell cultures by 78% (95% confidence intervals: 45–119%) (Figure 3A); there was a significant BUT concentration-dependent trend in ATP release (*p* < 0.001). The reported BUT effect was significantly attenuated when the cells were pre-incubated for 30 min with BAPTA-AM 50 μM, suggesting at least partial cytosolic Ca^2+^-dependent mechanism (Figure 3C). On the other hand, BUT in concentrations of up to 5 mM had no significant effect on Glu release from astrocytes (Figure 3B).

IPA produced a significant effect on neither ATP nor Glu releases from the tested cells, both following a 10-min treatment (Figure 3A,B) and after 24-h pre-incubation (Figure 3D,E).

## 3. Discussion

An appropriate gut microbiota composition and the intake of specific probiotic products allow for the body’s supply with bacterial metabolites such as BUT and IPA [10,11,49]. After being absorbed into the bloodstream, BUT and IPA penetrate the blood–brain barrier [11]. The metabolites are known to exhibit beneficial effects on the CNS’s components [14,15,16,17,18,19,20,21,23,25] including the astrocytes [22], which substantially contribute to the CNS’s function [26]. Until now, however, it was not known whether the metabolites modify astrocytic gliotransmitter release, the process important in cognition and mood regulation [27]. The present study is the first to demonstrate that BUT, at high CNS concentrations, but likely not IPA, increases ATP release from human astrocyte cultures in a cytosolic Ca^2+^-dependent manner. The results help to develop understanding of the mechanism of the effects of beneficial gut microbiota metabolites on the CNS function and in neuropsychiatric treatment.

Impaired ATP release from astrocytes located in the prefrontal cortex and the hippocampi has been suggested to cause major depression [31]. Mice that were susceptible to chronic social defeat were found to present with about 20% lower ATP abundance in their brains as compared with controls. Moreover, the specific blockade of the cytosolic Ca^2+^-dependent release of ATP from astrocytes in these mice caused depressive-like symptoms that could be rescued by the administration of ATP or the 2.5-fold astrocyte-specific activation of ATP release [34]. Concordantly, fluoxetine, a clinically used antidepressant medication, increased the ATP gliotransmission in the mouse model, and another molecular Ca^2+^-related disruption of astrocyte-specific ATP release impeded fluoxetine-induced anti-depressive action as examined in the tail-suspension test [50]. Moreover, the blockade of Ca^2+^-independent ATP release from astrocytes was found to cause neuronal synaptic dysfunction and depressive-like symptoms in mice that were reversed by ATP supplementation [51]. Notably, electroconvulsive and sleep-deprivation clinical therapies for depression were also reported to induce ATP release from astrocytes [52].

In light of the above-mentioned evidence, the BUT-induced Ca^2+^-mediated release of astrocytic ATP reported in our study appears to be an important factor contributing to the anti-depressive effects of BUT. These antidepressant-like actions of BUT were reported in several animal models [14,15,16,17,18,19,20,21], but have never been attributed to increased ATP release from astrocytes. As such, our study provides a novel mechanistic insight into BUT’s biological properties. This may further endorse specific gut-microbiota-targeted interventions in the adjunctive treatment of depression and support the rationale for testing BUT itself as potential antidepressant in clinical trials, which has now started to take place [53,54].

To assess the clinical relevance of the reported ATP-releasing effect, the critical question is whether the tested BUT concentrations are reached in the brain. In the human brain, a 0.02 mM concentration of BUT was reported [55]; however, it is not known whether the brain samples were of sufficient quality in that study to reliably represent BUT concentrations, which are unstable in aqueous solutions [56], and the characteristics of the brain donors were not reported [55]. In mice orally supplemented with BUT-producing *Clostridium butyricum* probiotic, BUT concentrations in the brain were in a range of 0.4–0.8 mM [57,58]. Despite scarce data, it may be generalized that brain levels of BUT may be highly variable, being dependent on the substrate availability, gut microbiota composition, and intestinal physical conditions [49], not to mention the ability of BUT to be absorbed and distributed into the brain [59]. The concentrations of ≥1 mM (which were effective in the present study) might be naturally achieved in astrocyte surroundings at most temporarily. However, such levels may be reached in vivo if a specific probiotic preparation is administered or likely during oral BUT supplementation; in fact, high daily doses of BUT are applied in animal studies (0.4–1.2 g/kg body mass) [15,16,17,20] and clinical trials (about 6 g) [60,61] to reveal CNS-relevant outcomes.

Interestingly, in our study, IPA demonstrated no significant effect on ATP release in either short- (10 min) or long-term (24 h) treatments. The latter incubation time was additionally applied due to a putative IPA mechanism of action dependent on AhR-mediated gene-transcription regulation [11]. Based on our insignificant results, it may be hypothesized that the AhR expressed in astrocytes may not be coupled with intracellular Ca^2+^ signaling [40,62] and not involved in ATP gliotransmitter release.

Insignificant results for Glu release obtained under both BUT and IPA influences require some discussion in light of the effect reported for control compounds. In the applied astrocyte cell line model, only the incubation with ionomycin resulted in a significant rise in Glu release, consistently with the fundamental report in this topic by Parpura et al. [63]. However, histamine—in contrast with the research by Kárpáti et al. [48]—failed to induce Glu release. Similarly, cytosolic Ca^2+^ chelator, BAPTA-AM, did not affect baseline Glu as expected [47], but only ionomycin-induced release. All of this data may suggest that the mechanism of Glu release in the examined astrocyte cultures occurs in response to high cytosolic Ca^2+^ levels which may only result after opening the cytosol to extracellular space and intracellular stores [46], but not physiologically increased intracellular Ca^2+^ levels resulting from the activation of the PLC-IP_3_ cascade pathway [48]. Meanwhile, the baseline Glu release may be Ca^2+^-independent [27]. These findings might further imply relative insensitivity of the Glu exocytotic machinery to Ca^2+^ in the tested cells. It is noteworthy that the human cortical astrocyte cells used in the present study were of fetal origin, obtained from a 19-week-old fetus. Vesicular glutamate transporters that are involved in Ca^2+^-dependent Glu gliotransmission may be expressed in delay during the prenatal development, as reported in the rats [64]; this may provide some possible explanation for the reported phenomenon of Glu release. Ultimately, the obtained insignificant results for Glu astrocytic release following BUT and IPA treatments should be regarded as inconclusive due to the discovered limitation of the model.

Our study presents another limitation as well. Firstly, it was carried out in a single cell-line culture, and—although it showed revealing results supported by multiple control-compounds treatments—it requires confirmation in another astrocyte model, also with regard to the discussed abovementioned Glu-release uncertainty. Secondly, it would be valuable to further explore the mechanism of BUT-induced ATP release to understand which molecules mediate the intracellular signaling leading to the effect; moreover, the question remains of how the effect influences adjacent neurons and brain function, and this could be examined by cell co-cultures and in pre-clinical models. Thirdly, the reported effect may not be specific to BUT, and other SCFA or similar compounds, which share FFAR-mediated mechanisms of action [10], may produce comparable astrocytic ATP releases. Finally, before BUT supplementation is widely recommended, the safety of its high doses should be clarified, as a controversy persists on whether BUT protects or promotes the development of colon cancer [65] and obesity [66]. Despite the limitations, our preliminary study may provide a novel mechanistic explanation for the beneficial effect of BUT on the CNS function.

## 4. Materials and Methods

### 4.1. Reagents

BUT, IPA, histamine, ionomycin, 1,2-bis(2-aminophenoxy)ethane-N,N,N′,N′- tetraacetic acid tetrakis(acetoxymethyl ester) (BAPTA-AM), 3-(4,5-dimethylthiazol-2-yl)- 2,5-diphenyltetrazolium bromide (MTT), dimethyl sulfoxide (DMSO), trypsin-EDTA solution, NaCl, KCl, MgCl_2_, CaCl_2_, glucose, and 4-(2-hydroxyethyl)-1-piperazineethane- sulfonic acid (HEPES) were purchased from Sigma-Aldrich (Saint Louis, MO, USA). Dulbecco’s Phosphate Buffered Saline was obtained from Biowest (Nuaillé, France). Astrocyte Medium, Astrocyte Growth Supplement, fetal bovine serum (FBS), penicillin/streptomycin solution, and poly-L-lysine were obtained from ScienCell Research Laboratories (Carlsbad, CA, USA).

### 4.2. Human Astrocyte Cell Culture

The study was performed with the use of a commercially available astrocyte cell line isolated from human cerebral cortex (ScienCell Research Laboratories, cat no. 28523). The cell culture was maintained at 37 °C in a humidified atmosphere of 5% CO_2_, in dishes coated with poly-L-lysine. The cells were grown in Astrocyte Medium supplemented with 2% FBS, 10% Astrocyte Growth Supplement, and 1% penicillin/streptomycin solution. Pure Astrocyte Medium was used as the experimental medium. The astrocyte culture was maintained according to the protocol recommended by ScienCell Research Laboratories.

### 4.3. Cell Viability MTT Test

In order to assess whether BUT and IPA are non-toxic to the cultured astrocytes, cell viability was evaluated using the colorimetric MTT assay. The cells were seeded onto a 96-well plate to a final density of 1.5 *×* 10^4^ cells/well. After 24 h of culture, the cells were exposed to BUT (up to 25 mM) and IPA (up to 0.25 mM) for the next 24 h. After the incubation time with the compounds, MTT solution was added to the cell culture for another four hours to let the mitochondrial succinate-tetrazolium reductase system convert yellow tetrazolium MTT into purple formazan. The absorbance was measured at λ = 570 nm by BioTek EL ×800 microplate reader (BioTek; Winooski, VT, USA); the value was proportional to the number of viable cells. The viability was calculated as a proportion of the absorbance of investigated samples to the absorbance of control samples (cells not treated with BUT and IPA).

### 4.4. ATP and Glu Release Dynamics

For the assessment of ATP and Glu release from astrocytes in the function of time, the cells were seeded onto a 96-well plate to a final density of 1.5 *×* 10^4^ cells/well for 24 h. Afterwards, the cells were washed and supplied with fresh experimental medium (in case of ATP assessment) or HEPES-buffered solution (HBS; 120 mM NaCl, 5 mM KCl, 1 mM MgCl_2_, 1.5 mM CaCl_2_, 10 mM glucose, 25 mM HEPES; pH = 7.4; in case of Glu assessment). After the incubations lasting 2, 5, 10, 15, or 30 min, the supernatants were collected and subjected for the measurands evaluation. For ATP, the luminescence-based test “ATP Detection Assay Kit—Luminescence” (Cayman; Ann Arbor, MI, USA; cat no. 700410) was applied according to the protocol recommended by the producer, and luminescence was assessed with the use of a Synergy H1 plate reader (Biotek; Winooski, VT, USA). For Glu measurement, the fluorescence-based test “Glutamate Assay Kit (Fluorometric)” (Abcam; Cambridge, UK; cat no. ab138883) was used according to the protocol recommended by the producer, and fluorescence (excitation λ = 550 nm, emission λ = 590 nm) was assessed with the use of a Victor X4 multilabel plate reader (PerkinElmer; Waltham, MA, USA).

### 4.5. Effect of the Control and Tested Compounds on ATP and Glu Release

For the assessment of ATP and Glu release in response to positive controls (histamine [48] and ionomycin [46,63]) and a negative control (BAPTA-AM [47]), as well as under the influence of BUT and IPA treatments, the cells were seeded onto a 96-well plate to a final density of 1.5 *×* 10^4^ cells/well. After 24 h, the cultures were washed and exposed to the control substances (histamine 100 μM or ionomycin 5 μM), and the tested compounds (BUT range of 0.2–5 mM and IPA range of 0.001–0.1 mM) dissolved in fresh experimental medium or HBS buffer (for ATP and Glu assessment, respectively) or with no addition of these substances. The exposition time was 10 min. Then, the supernatants were collected for the immediate analysis of the measurands that was carried out as described above. Before the cell culture wash, the cells were optionally pre-incubated with BAPTA-AM (cytosolic Ca^2+^ chelator) for 30 min or IPA for 24 h.

### 4.6. Data Analysis

All of the experimental procedures to examine BUT’s and IPA’s effects on the ATP and Glu concentrations in the supernatants were performed in three biological replicates, and each such experiment was independently repeated at least three times (nine samples in total for each experimental condition). Each individual supernatant sample was analyzed in two technical replicates, which were averaged before further analysis. One-way analysis of variance (ANOVA) with Dunnett’s post-hoc test was used to test the null hypotheses of no difference in the mean concentrations of measurands between the cells treated with specific concentrations of the tested compounds in comparison with those untreated. The contrast analysis was optionally used to evaluate the concentration-dependent trend in the effects. Statistical significance was set at α = 0.05. STATISTICA software version 13.3 was used in the analysis (Statsoft; Tulsa, OK, USA).

## 5. Conclusions

This is the first report to preliminarily demonstrate BUT—in high brain concentrations—to increase ATP gliotransmitter release from the cultured human astrocytes in a cytosolic Ca^2+^-dependent manner; IPA was found to not significantly affect ATP release. The findings appear to provide a novel mechanistic explanation for the beneficial effect of BUT on CNS function. This may further endorse testing of BUT-producing gut-microbiota-targeted interventions and BUT supplementation as potential antidepressants. The results of BUT’s and IPA’s effects on Glu release remain inconclusive. The reported findings require confirmation and further testing in basic and pre-clinical settings.

## Data Availability

The data presented in this study are openly available in the Mendeley Data repository at digital object identifier: 10.17632/f9y2dcfb99.1.
